# Progress in Glaucoma Management in the Era of Value-Based Healthcare

**DOI:** 10.3390/jcm15010138

**Published:** 2025-12-24

**Authors:** Adèle Ehongo

**Affiliations:** Cliniques Universitaires Saint Luc, Avenue Hippocrate 10, 1200 Bruxelles, Belgium; adele.ehongobidime@saintluc.uclouvain.be; Tel.: +32-27641950

**Keywords:** value based healthcare, VBHC, glaucoma, outcome, OCT, SLT, minimally invasive glaucoma surgery, indicator, effectiveness, medications

## Abstract

Medical care is increasingly evolving towards patient-centered medicine, which is at the heart of the value-based healthcare (VBHC) model. The VBHC model simultaneously prioritizes better health outcomes while optimizing costs. Its application therefore requires the use of quantitative indicators to assess the performance of healthcare systems. At the same time, in glaucoma, minimally invasive procedures are expanding therapeutic options, gaining popularity and establishing themselves as an attractive alternative to traditional, riskier glaucoma surgeries. These safer procedures are increasingly enabling early intervention in the care pathway of glaucoma patients. However, their cost-effectiveness remains to be determined. This work aims to analyze the impact of the current approach to glaucoma management through the VBHC model and to discuss performance indicators that allow for effective evaluation.

## 1. Introduction

The increase in chronic diseases, combined with the aging population, is forcing health organizations to spend ever more without improving outcomes.

In ophthalmology, glaucoma is among the four conditions that alone account for 70% of consultations and costs in referral centers [1]. Furthermore, a sharp increase in healthcare costs related to glaucoma treatment is expected, as the prevalence of this leading cause of irreversible blindness [2] increases with age. Its global prevalence is estimated to reach 111.8 million people by 2040 [3].

Faced with limited resources, healthcare systems are transforming to improve their cost-effectiveness. They are thus shifting from the quantity of services offered (volume-based) to the quality of care (value-based) referred to as the Value-Based Healthcare (VBHC).

Introduced by Porter [4], the VBHC is a patient-centered approach to care [4] aiming to improve the most important health outcomes for patients throughout their care pathway, while optimizing health system resources and costs, both for the patient and for society.

Since it is defined as the health outcomes achieved by patients, relative to the cost of care (outcomes/cost), value can thus increase by improving outcomes, minimizing costs, or both [5]. From the patients’ perspective, value is assessed using indicators such as patient-reported outcome measures (PROMs) and patient-reported experience measures (PREMs) [6].

This work analyzes some recent advances and developments in the field of glaucoma, highlighting their potential to foster a transition to VBHC. From diagnostic assessment to glaucoma treatment, the focus is on reducing healthcare costs through the avoidance of unnecessary procedures and improved care coordination, as well as improving outcomes and patients’ quality of life. Finally, the complex issue of key performance indicators for VBHC is addressed.

## 2. Value-Based Healthcare and Glaucoma Diagnosis

Through the perspective of VBHC, the patient’s journey from the discovery of the first signs suggestive of glaucoma to the final diagnosis is not so straightforward, especially when the eye pressure is normal.

This specific type of glaucoma, called Normal Tension Glaucoma (NTG), first requires ruling out its potential differential diagnoses. Interestingly, it has recently been highlighted that Optical Coherence Tomography (OCT) can easily eliminate two masquerades of NTG at the initial clinical evaluation [7,8]. The concordance between visual field defects [8,9] and the location of the confounder [8,10,11] speeds up the diagnosis, while it has been reported that 40% of patients eventually diagnosed with buried optic discs drusen in a tertiary center had first been diagnosed as NTG [8].

OCT thus makes it possible to avoid additional examinations, particularly neuroimaging [12], to rule out papilledema [8,12] or an optic disc tumor [7]. Most importantly, OCT has demonstrated a higher detection rate for buried optic disc drusen than B-mode ultrasound, which until now had been the gold standard [13,14].

Overall, careful examination of the optic disc using OCT, a gold standard technique in ophthalmology, meets many VBHC criteria. It improves efficiency by reducing healthcare costs through the avoidance of unnecessary examinations [12] and shortens the patient’s care pathway before the final diagnosis.

## 3. Value-Based Healthcare and Non-Surgical Treatment of Glaucoma

To prevent additional damage, Intraocular Pressure (IOP) reduction is currently the only proven glaucoma treatment [15]. This reduction can be achieved by many ways, including eye drops, laser, filtering surgeries and Minimally Invasive Glaucoma Surgery (MIGS) [16,17,18,19].

Interestingly, the place of Selective Laser Trabeculoplasty (SLT) has recently changed in the therapeutic arsenal of glaucoma [17] thanks to the results from the Laser in Glaucoma and ocular Hypertension Trial (LiGHT). The following elements of the LiGHT study fall within the VBHC framework.

### 3.1. Patients’ Outcomes

This multicentric Randomized Controlled Trial (RCT) first showed that at 3 years, SLT as a primary treatment is at least as effective as eye drops [17] in lowering IOP for Ocular Hypertension (OHT) and mild or moderate Open Angle Glaucoma (OAG). It also demonstrated a delay in the need for surgery [17].

Subsequently, long-term results revealed that SLT is a safer treatment than eye drops for OAG and OHT [20]. Furthermore, SLT provides better long-term disease control than initial drop treatment, thus reducing the need for incisional glaucoma and cataract surgery over 6 years [20].

Finally, when used for escalation or shift, SLT allows both the reduction in IOP and the number of IOP-lowering medications in eyes that have received IOP-lowering drugs as first-line treatment [21].

### 3.2. Patients’ Costs

Interestingly, from an ophthalmology cost perspective, there was a 97% probability of SLT as first treatment being more cost-effective than eye drops first at a willingness to pay £ 20,000 per quality-adjusted life-year gained (QALY), with a reduction in ophthalmology costs of £458 per patient [17].

### 3.3. Patients’ Quality of Life (QoL)

This dimension was explored using health-related QoL (HRQoL) (as measured using the EuroQol-5 Dimensions, five-level version (EQ-5D-5L) questionnaire). The study showed no significant difference at 3 years between the SLT, and eye drops groups for disease-specific HRQoL [17].

At 6 years, the SLT arm showed better Glaucoma Symptom Scale Scores than the drops arm, while both arms were comparable for the EuroQoL EQ-5D 5 Levels, Glaucoma Utility Index and Glaucoma QoL-15 [20].

### 3.4. Long-Term SLT Value

As presented in the outcomes section above, SLT as first-line treatment reduces the need for incisional glaucoma and cataract surgery over 6 years [20]. The need for surgery was not eliminated when SLT was used as a secondary treatment [21].

Overall, current data show that as first-line therapy, SLT is safer, more reproducible and more cost-effective than eye drops for the treatment of OHT and OAG [17,20]. It reduces the need for glaucoma and cataract surgery [20]. When used as second-line therapy, it allows for better control of IOP and a reduction in the number of eye drops required [21], thus meeting several criteria for VBHC.

## 4. Early Interventions in Glaucoma

The 30-day unplanned readmission rate is recommended by the World Health Organization as an indicator of health system performance and provides a standardized quantitative assessment of certain postoperative complications [22]. Using this quality-of-care indicator, Crozet showed that glaucoma filtering surgery is the type of ophthalmic surgery associated with a high risk of unplanned readmission at 30 days, accounting for 22% of all readmissions [23]. Among elective surgeries, trabeculectomy had even the highest unplanned readmission rate, averaging 11.6% [23].

Although considered the gold standard in glaucoma surgery, trabeculectomy is now relegated to the last resort in advanced glaucoma patients, requiring low target IOPs, due to its high complication rate [24]. The study comparing the tube to trabeculectomy reported a respective rate of 34% and 27% for early and late postoperative complications in the trabeculectomy group after five years of follow-up [25].

Gradually, MIGS are emerging, offering promising alternatives at an earlier stage of the disease [19,26].

It should be noted that the frequency of co-occurrence of glaucoma and cataracts increases with age. It is estimated that 20% of patients who have undergone cataract surgery take medication to reduce IOP [27]. This raises the question of a comprehensive approach to managing this comorbidity, as it is essential to treat the patient as a whole and not just one of these conditions, which is consistent with VBHC.

Furthermore, MIGS were shown to potentialize the IOP-lowering effect of phacoemulsification (phaco) [28,29,30,31,32].

Thus, in patients with cataract associated with mild or moderate OAG or OHT, phaco combined with MIGS (phaco–MIGS) is an option that is generating increasing interest [29,30]. Phaco–MIGS therefore represents an effective solution for better IOP control and may even reduce the number of eye drops required when phaco is considered in a patient with OAG [28,29,30,31].

As standalone procedures, MIGSs are also effective and reduce the burden associated with eye drops. The benefit of eliminating eye drops in glaucoma treatment is summarized in Table 1.

## 5. Value-Based Healthcare and MIGSs

### 5.1. MIGSs Outcomes

MIGS are quick procedures, known to be minimally invasive [19,33,34,35,36], allowing for faster recovery time [28,35,36,37,38,39]. 

These techniques are associated with low and benign complication rates, primarily hyphemas or IOP spikes [19,34,36,37,40] with no cases of infection or loss of best corrected visual acuity reported [19].

Except for MIGS with a filtering bleb, as the Xen Stent, where revision is common (23.3%), to improve the success rate [19,41], MIGs are effective in maintaining physiological levels of IOP and are therefore indicated when the desired target IOP is not low [19,36].

MIGS, particularly those that preserve the conjunctiva [42,43] constitute, therefore, an intermediate step before considering more invasive options. The longest follow-up duration published for some of the MIGS reaches 10 years (Table 2).

### 5.2. Costs, Accessibility and Adherence for MIGSs

Given the increasing number of MIGS devices, it is crucial to have standardized comparison tools helping surgeons and other stakeholders in decision making. Many studies are retrospective, with no controls [33,37,44], involve small samples [38,39] and have limited follow-up, which justifies RCT to overcome these drawbacks. To this end, a common indicator allowing for the most accurate direct comparisons remains an unmet need.

The impact of MIGS on the reported QoL of the patients, as well as their PROMs and costs, also remain to be studied.

## 6. MIGS and Performance Indicators

### 6.1. Result of a Procedure in Terms of Eye Drops Eliminated

As current data regarding the cost-effectiveness and safety profile of MIGS and phaco–MIGS are primarily derived from non-comparative studies [19,29,30]; high-quality RCTs using relevant endpoints are warranted.

Generally, the standard criteria for evaluating the success of glaucoma interventions refer to qualified and complete success. These are defined as an IOP below 21 mmHg and an IOP reduction of ≥20% compared to preoperative IOP under drug therapy, with or without adjuvant medical treatment, respectively [19].

However, the effectiveness of MIGS is limited to achieving a physiological IOP [19,43].

Thus, MIGS primarily aims to address the needs of patients who do not require invasive surgery but do not respond well to eye drop treatment or for whom a dosage reduction would be beneficial. Therefore, the number of IOP-lowering drugs eliminated [29,30] is also relevant as an evaluation criterion.

Furthermore, after MIGS interventions, the IOP reduction effect and its duration differ from patient to patient, as does the reduction in the number of medications [19,29,30,37,44].

Since each patient can benefit from a reduction in the number of eye drops required, over a variable period of time, the benefit for each patient can be quantified by multiplying this duration in years (Y) by the number of Medications (M) eliminated: (MY). Table 3 presents the combined result of the number of eye drops eliminated and the elimination duration.

The MY indicator thus provides, by a single value, for a given patient, the combined information on the number of drops eliminated and the duration of their elimination.

A patient can have a MY of 1 if they have benefited from a reduction in one medication (Med) for one year or a reduction in two Meds for six months.

Because the benefit can vary over time for the same patient, this method allows for precise quantification of the total benefit of the intervention for each patient by adding the different segments of MY. Each MY segment corresponds to the product of the number of Med reduced and the duration of that reduction.

Example 1: Let us suppose a patient benefited from a reduction in two Meds after the procedure for one year. Subsequently, their condition required the addition of an active ingredient, which stabilized their IOP for five years. After six years, the total benefit of the procedure will be 7 MY, calculated as follows: (2 Meds × 1 year) + (1 Med × 5 Years) = 2 + 5 = 7 MY.

This means that after 6 years, the intervention carried out allowed this patient to preserve his ocular surface by an average of 7/6 = 1.17 molecules per year.

Example 2: One patient included in a study assessing the efficacy of a MIGS procedure that lasted 5 years in total. He had 3 Meds prior to the MIGS procedure. After the intervention, the patient achieved the target IOP without Med for 1 year. Then, one additional Med was required to achieve the target IOP for 1.5 years. Finally, a second additional Med was required to achieve the target IOP until the end of the study. Here is the total benefit of the procedure for this patient at the end of the study in terms of Meds reduction:

We have 3 segments to add up. The total MY for the 5 years is (3 Meds × 1 year) + (2 Meds × 1.5 years) + (1 Med × 2.5 years) = 8.5 MY for 5 years.

First segment: The patient did not use the previous three eye drops for 1 year. MY = 3 × 1 = 3 med·years.

After reintroduction of the first eye drop, the patient did not need two of his previous eye drops for 1.5 years, compared to the preoperative period. MY = 2 ×1.5 = 3 med·years.

Finally, their target IOP was maintained until the end of the study after reintroduction of the second eye drop. The patient therefore had one less Med compared to the situation before the procedure for 2.5 years (5 years − (1 + 1.5) = 2.5 years). MY = (1 × 2.5) = 2.5 med·years.

### 6.2. Generalization Equation

Let us analyze the case of a patient who was taking d kind of drops before an intervention for glaucoma.

We denote by $A_{i}$ the number of years during which the patient took i kind of drops per day after intervention. We can therefore construct the vector

$$A=\left(A_{0},A_{1},A_{2},\ldots ,A_{n}\right)$$

which represents, in order, the number of years during which the patient took i kind of drops per day, for i ranging from 0 to n. In practice, no patient is prescribed more than 4 different kinds of drops per day, so the maximal value of n should be 4.

We define the benefit b of the intervention as the average number of kind of drops avoided per day after intervention. Hence, b can be written as

$$b=\frac{\mathrm{number}\;\mathrm{of}\;\mathrm{kind}\;\mathrm{of}\;\mathrm{drops}\;\mathrm{avoided}\;\mathrm{per}\;\mathrm{day}}{\mathrm{total}\;\mathrm{number}\;\mathrm{of}\;\mathrm{days}\;\mathrm{recorded}}$$

This quantity can be expressed mathematically using A and d as

$$b=\frac{\left(d-0\right)A_{0}+\left(d-1\right)A_{1}+\left(d-2\right)A_{2}+\cdots +\left(d-n\right)A_{n}}{A_{0}+A_{1}+A_{2}+\cdots +A_{n}}$$

which can be rewritten as

$$b=\frac{\sum_{i}^{n}\left(d-i\right)A_{i}}{\sum_{i}^{n}A_{i}}$$

with n in practice limited to 4 as stated above.

Indeed, $\left(d-i\right)$ represents the number of kinds of drops avoided relative to the initial prescription of d kind of drops when the patient is taking i kind of drops per day. This term is multiplied by the duration $A_{i}$ during which the patient took i kind of drops per day. Dividing this result by the total observation period yields an average expressed in kind of drops avoided per day.

Illustration:

- Let us consider a patient x who was taking d=3 kind of drops per day before an intervention for glaucoma.

After the intervention, patient x lived for 5 years without any drops ${(A}_{0}=5)$, before being prescribed 1 kind of drop per day again, which he took for 6.3 years ${(A}_{1}=6.3)$. Subsequently, the prescription was increased to 2 kinds of drops per day which he has been taking for 1.7 at the time of observation ${(A}_{2}=1.7)$.

We thus have

$$A=\left(A_{0},A_{1},A_{2},A_{3},A_{4}\right)=\left(5,6.3,1.7,0,0\right),\sum_{i=0}^{n}A_{i}=13$$

The benefit is therefore

$$\begin{array}{cc} b & =\frac{\left(3-0\right)\times 5+\left(3-1\right)\times 6.3+\left(3-2\right)\times 1.7}{13}=\frac{29.3}{13}\\ & =\;2.25\;\mathrm{kind}\;\mathrm{of}\;\mathrm{drops}\;\mathrm{avoided}\;\mathrm{per}\;\mathrm{day}\;\mathrm{since}\;\mathrm{the}\;\mathrm{intervention} \end{array}$$

- Patient y was initially taking d=2 kind of drops per day before another intervention. We recorded the following post-intervention evolution

$$A=\left(1,5,0,0,0\right),\sum_{i}^{n}A_{i}=6$$

The benefit is therefore

$$\begin{array}{cc} \;b & =\frac{\left(2-0\right)\times 1+\left(2-1\right)\times 5}{6}=\frac{7}{6}\\ & =1.17\;\mathrm{kind}\;\mathrm{of}\;\mathrm{drops}\;\mathrm{avoided}\;\mathrm{per}\;\mathrm{day}\;\mathrm{since}\;\mathrm{the}\;\mathrm{intervention} \end{array}$$

Therefore, we can conclude that patient *x* experienced a greater benefit than patient *y* after their respective interventions.

### 6.3. Usefulness of MY Indicator

Similarly to the QALY, which assesses the value of a medical intervention by combining both quantity and quality of life [45,46], the MY indicator assesses the overall benefit of an intervention in terms of reduction in eye drops over a defined period.

**Figure 1:**
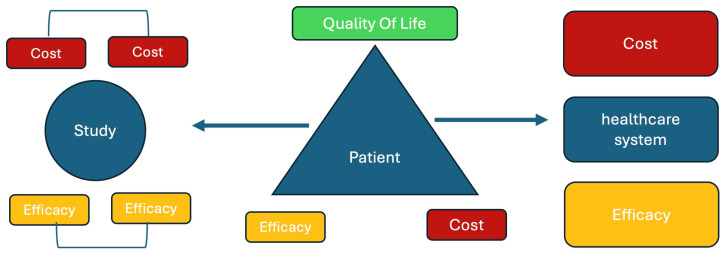
The potential usefulness of MY.

This approach, applied to a given number of patients participating in a study on a MIGS intervention, will make it possible to quantify the long-term effectiveness of the MIGS in question. By determining the number of MY gained for each patient over the entire duration of the study and summing the MY of all patients, the total number of MY for this intervention in the study is obtained. Dividing this number by the total number of patients involved gives the average benefit of the intervention per patient: average MY per patient for the duration of the study. Figure 1 illustrates the usefulness of MY in assessing value from the perspective of the patient, studies and decision-makers.

MY can help assess the cost-effectiveness of a procedure for all stakeholders in the healthcare ecosystem. It can be used to analyze different VBHC criteria at different levels.

Converted on an annual basis, by dividing this MY by the total duration of the study, the average MY per patient per year can then serve as a unit of comparison for the long-term effectiveness of the different MIGS procedures.

To account for the variable efficacy of different classes of Meds, RCTs could begin with standardization of treatments. Subsequently, to provide a reliable basis for comparison during long-term follow-up, the protocol should also define the order of reintroduction of the different classes of molecules to achieve the target IOP.

For each patient, the cost, effectiveness and effect on QoL can also be quantified using the MY as basis.

Finally, the healthcare system can leverage MY to assess the effectiveness and cost of a procedure while making its decision.

The MY concept, having just been introduced in this article, will need to be implemented concretely. Its main advantage lies in the fact that it offers a unique and fundamental unit of comparison, applicable to patients, funders, clinicians and researchers, and likely to be used in high-quality long-term studies.

It allows us to assess the benefit of any type of intervention that eliminates or reduces the need for eye drops, including SLT and filtering surgical procedures.

Its main limitation is that the MY indicator cannot be calculated a priori, which is inherent to clinical observations. Indeed, for a given patient, we cannot accurately predict in advance the value of their postoperative IOP, nor the duration of the initial postoperative benefit obtained, nor the long-term evolution of this benefit. Nevertheless, this indicator remains highly relevant and would benefit from being implemented in practice.

## 7. VBHC Model and Glaucoma Clinic Organization

Given its progressive nature, the goal of glaucoma treatment is to stabilize the disease. This therefore requires regular follow-up examinations. These include structural examinations such as optic nerve imaging or OCT, and functional examinations such as visual field testing. The acquisition of these examinations is carried out by technicians. The technicians and specialists together constitute an integrated practice unit. The acquisition devices are equipped with software that also allows for progression analysis. Based on the VBHC framework, Table 4 summarizes the current state of care of this clinic and potential improvements.

## 8. Patient Satisfaction

Most ophthalmologic interventions are especially cost-effective by conventional standards [45].

With the aim of placing the patient at the center, by aligning care with their personal goals and values, VBHC involves defining cost, quality of care and value indicators that are meaningful to the patient.

However, from the patients’ point of view, quality of care measures and perceived value of care are two indicators that are difficult to define [46].

It is important to note that, given its chronic and progressive nature, the most important indicators for patients, which then allow for relevant analysis, monitoring and treatment, evolve according to the stage of glaucoma.

Glaucoma is unique because it is asymptomatic in its early stages, a period during which the patient is mainly bothered by the potential side effects of eye drops [47,48,49] and ocular surface disease [50], which leads to non-compliance with treatment [51], patient dissatisfaction [52], and an increased risk of progression [53]. Interventions such as SLT which showed its superiority over eye drops should then be preferred [17,20,21].

For patients using eye drops, PROMS or PREMS should include an assessment of ocular surface diseases [53].

When phaco is planned, this would be the ideal opportunity to perform an additional MIGS intervention, allowing, in many cases, to reduce the load of eye drops [28,29,30,31].

In advanced stages, glaucoma patients suffer from a disability that interferes with their daily activities. Treatment becomes more difficult, and surgery riskier, because the target IOP for advanced stages of glaucoma is set at low values, necessitating filtering surgeries such as trabeculectomy [18]. PROMS or PREMS questionnaires should then include the assessment of disability.

However, collecting PROM and PREM data faces logistical obstacles regardless of the method. Electronic questionnaires would minimize the risk of errors. However, some patients, for various reasons (procrastination, forgetfulness…), will not complete these questionnaires, resulting in a lower response rate. Digital illiteracy can also be a significant obstacle for certain segments of the population [54]. Filling out forms manually will result in a higher response rate but will increase the risk of data entry errors. Providing oral versions would allow us to reach patients who are unable to write. However, the workload and processing time are increased for manual or oral procedures. The time factor is even more important given the time required for the clinical management of glaucoma [55]. In general, it is advisable to use these different data collection methods to promote fairness.

It was highlighted that participation in the implementation of PROMs was much lower among patients from more disadvantaged socioeconomic and educational backgrounds, who tend to be the most severely affected by eye diseases [56].

The lack of appropriate and sensitive measurement tools for patients’ PROMs in ophthalmology is illustrated by the results of the LiGHT study showing that the only questionnaire that revealed a difference in QoL between the group treated with SLT and the group treated with eye drops was the Glaucoma Symptom Scale Score [17,20,21].

Interestingly, a recent systematic review showed that three questionnaires in a research setting are the most validated in the group of glaucoma patients [6]. However, the need for further studies was highlighted because identifying a single optimal questionnaire for clinical use is challenging due to the limited number of reports on the interpretability, responsiveness and feasibility of current tools [6]. Thus, one of the priority areas for improvement concerns PROM and PREM [57].

## 9. Summary

In practice, integrating close and careful analysis of OCT before moving to complimentary exams in the assessment of NTG suspects fits with the VBHC as it redesigns the workflow efficiently. It shortens the patient care journey before the diagnosis and reduces overall healthcare costs [7,8,9].

Beyond its effectiveness, SLT both preserves the QoL of patients and delays the need for further surgery, contributing to patient-centered, better outcomes [17,18,19,20].

In patients with OHT or mild to moderate OAG who are candidates for cataract surgery, phaco–MIGS techniques contribute to comprehensive and coordinated care, allowing for better control of IOP and even a reduction in antiglaucoma medications [19,29,30]. This results in an improved QoL for the patient.

Calibrated and standardized MIGS techniques, which allow for reproducibility and facilitate comparisons [26], are of interest. These characteristics are valuable because one of the challenges of VBHC lies in the need to develop standardized measurement tools that enable comparisons. Implant-free techniques are interesting, as are conjunctival-preserving techniques, which retain the possibility of subsequent interventions for the management of glaucoma [35].

Performance indicators remain the main challenge for VBHC, particularly the development of measures allowing comparative studies and RCTs [46].

## 10. Conclusions

Ophthalmology, by its very nature, already falls under the value-based healthcare approach with the majority of ophthalmic interventions being cost-effective according to conventional standards.

Current efforts focus on defining, developing, improving, and standardizing performance indicators from the patients’ perspective and for MIGS comparisons.

The MY indicator is a method for evaluating the cost-effectiveness of glaucoma interventions, taking into account both the number of eye drops reduced and the duration of this reduction. It measures the effectiveness of a technique in terms of reducing the number of active ingredients needed to achieve the target IOP.

It can be integrated into the care ecosystem by all stakeholders, starting with the patient. Its integration and improvement within the VBHC model would be beneficial for analyzing the drops reduction capacity of a procedure over a given period.

## Figures and Tables

**Table 1:** Added value of eliminating eye drops in the glaucoma healthcare landscape.

Target	Outcome	SLT	MIGS
Patients-outcomes improvement	Elimination of side effects	X	X
	Elimination of preservatives	X	X
	Elimination of compliance problems	X	X
	Preservation of the ocular surface	X	X
	Improved quality of life	X	X
	Slowing of progression	X	
	Delayed surgery	X	
	Allows for better results for filtering surgery by preserving the conjunctiva	X	X
Costs reduction for patients	Elimination or reduction in different types of drops	X	X
	Elimination of artificial tears for dry eyes favored by glaucoma treatment	X	X
	Elimination of unscheduled visits related to ocular surface disease	X	X
	Elimination of unscheduled visits related to allergic reaction	X	X
Costs reduction for healthcare system	Elimination of unscheduled visits related to ocular surface diseases	X	X
	Elimination of unscheduled visits related to allergic reaction	X	X
	Disability reduction (related to slowing progression and better adherence)	X	X
	Slowing of progression (will save money related to incremental treatment)	X	

X: criterion met by the procedure. Absence of X: criterion not met by the procedure. Abbreviation. SLT: Selective Laser Trabeculoplasty. MIGS: Minimally Invasive Glaucoma Surgery.

**Table 2:** Results at one year and at the longest follow up duration of main MIGS.

MIGS Target	Author, Year [Ref]	MIGS Type	LfD Reported	% of Eyes with IOP Reduction		% of Eyes with Drop Reduction	
				1 year	LfD	1 year	LfD
Trabecular bypass	Salimi, 2025 [37]	Hydrus	5	23.6%	25.8%	35.7%	21.4%
	Salimi, 2021 [44]	i-Stent	8	(17.7%)	26%	(53.6%)	18%
	Bendel, 2018 [40]	Trabectome	8	90%	20–23%	(1.73)	(1.13)
	Bektas, 2025 [33]	GATT	5	50–80%	30–40%	50–70%	50–70%
	Vasu, 2024 [36]	KDB	6	50–60%	25–38%	30.8%	>50%
	Berlin, 2022 [35]	ELT	8	80%-C	50%		(1.2)
Schlem’s canal	Kailani, 2025 [38]	Trab 360	10 (20)	70–80%	50–80%	“high” %	“high” %
	Kailani, 2025 [38]	OMNI	3	82%-C	70–90%		
	Beres, 2025 [34]	Ab Interno Canaloplasty	10	30–65%-C	23–30%	>50%	50%
	Amiri, 2025 [42]	Streamline	1	87.5%-Q		50%	-
*		i-Stent supra	-	-	-	-	-
Bleb forming	Rauchegger, 2024 [41]	XEN gel	3	40%-C	27.3%-C	62.8%	45.4%
	Batlle, 2021 [39]	PreserFlo	5	82.6%-C	82.6%	60%	61%

Abbreviations. C: complete success, Q: qualified success, LfD: longest follow-up duration, GATT: Gosnioscopy Assisted Transluminal Trabeculotomy, KDB: Kahook Dual Blade. ELT: Excimer Laser Trabeculostomy. * Suprachoroidal position.

**Table 3:** Combined result of the number of eye drops eliminated and the elimination duration.

		Number of Eye Drops Reduction (M)		
		1	2	3
Duration of the drop elimination (Y)	0.5	0.5	1	1.5
	1	1	2	3
	1.5	1.5	3	4.5
	2	2	4	6
	2.5	2.5	5	7.5
	3	3	6	18
	10	10	10	30

**Table 4:** Application of VBHC in the organization of glaucoma clinics.

Core Component of VBHC	Item	Actions Suggested
Care Organization into Integrated Practice Units	- Skilled and experienced technicians in capturing glaucoma data (visual field, OCT, photos); - Data acquisitions; - Glaucoma specialists.	- Increase the availability of skilled technicians. - Increase the availability of glaucoma specialists. - Have glaucoma-trained medical staff in all specialty centers. - Regular training update for staff. - Potentialize best acquisitions and develop easy to use devices/
Outcomes and Cost Measurement for Every patient	- Systematic tracking of clinical outcomes (visual field, OCT, IOP); - Appropriate costs documentation; - PROMs measurements.	- Develop appropriate tools for cost measurements for every patient. - Implementation of PROMs to be improved. - Implement how to overcome obstacles on PROM.
Move to Value-Based Payments	- Appropriate definition of difficulty in outcomes; - Appropriate definition of the clinical burden of care based on the stage of glaucoma disease.	- Explore the best way of developing and implementing means of achieving excellent outcomes over time, and not just for individual visits. - Stratify bundle payments based on the defined outcome level and disease stage.
Care Integration across the Continuum	- Care coordination from primary care to specialists; - Support services; - Referral process.	- Implementation of shared data platforms. - Improvement of support services. - Raise awareness among primary centers about the appropriate referral time to secondary centers.
Build Enabling IT Platforms	- Integrated softwares analyzing and predicting progression in the visual fields and OCT devices; - Electronic medical records; - Resources for appropriate recording.	- Implement digital systems facilitating the share of patients information. - Improve compatibility of existing softwares. - Increase the availability of electronic medical recorders. - Providing easy-to-use platforms. - Providing resources (technical, time, personnel, financial, etc.).
Empower Patients and Shared Decision Making (SDM)	- Patients clear information; - Patients’ involvement in glaucoma treatment choices (types of drops/ laser/ surgery).	- Develop and improve digital tools for patients’ education. - Improve and ease the use of PROMs and PREMs. Develop facilitators for this use. - Explore ways to overcome barriers to the use of PROMs.

## Data Availability

Not applicable.

## Abbreviations

The following abbreviations are used in this manuscript:

ELT	Excimer Laser Trabeculostomy
EQ-5D-5L	Euro Quality of Life-5 Dimensions, five-Level
GATT	Gonioscopy-Assisted Transluminal Trabeculotomy
HRQoL	Health-Related Quality of Life
IOP	Intraocular Pressure
KDB	Kahook Dual Blade
LiGHT	Laser in Glaucoma and ocular Hypertension Trial
Med	Medication
MIGS	Minimally Invasive Glaucoma Surgery
MY	Medication X Year
NTG	Normal Tension Glaucoma
OAG	Open Angle Glaucoma
OCT	Optical Coherence Tomography
OHT	Ocular Hypertension
Phaco	Phacoemulsification
phaco combined	Phaco-MIGS
PREMs	patient-reported experience measures
PROMs	patient-reported outcome measures
QALY	Quality-Adjusted Life Year)
QoL	Quality of Life
RCT	Randomized Controlled Trial
SLT	Selective Laser Trabeculoplasty
VBHC	Value-based healthcare
